# Transcranial Magnetic Stimulation Measures in the Elderly: Reliability, Smallest Detectable Change and the Potential Influence of Lifestyle Habits

**DOI:** 10.3389/fnagi.2018.00379

**Published:** 2018-11-27

**Authors:** Francis Houde, Sarah Laroche, Veronique Thivierge, Marylie Martel, Marie-Philippe Harvey, Frederique Daigle, Ailin Olivares-Marchant, Louis-David Beaulieu, Guillaume Leonard

**Affiliations:** ^1^Faculty of Medicine and Health Sciences, Université de Sherbrooke, Sherbrooke, QC, Canada; ^2^Research Centre on Aging, CIUSSS de l’Estrie – CHUS, Université de Sherbrooke, Sherbrooke, QC, Canada; ^3^Biomechanical and Neurophysiological Research Lab in Neuro-Musculo-Skelettal Rehabilitation, Université du Québec à Chicoutimi, Chicoutimi, QC, Canada

**Keywords:** transcranial magnetic stimulation, aging, elderly, lifestyle habits, chronic disease, reliability, smallest detectable change

## Abstract

**Background:** Transcranial magnetic stimulation (TMS) is a non-invasive technique that can be used to evaluate cortical function and corticospinal pathway in normal and pathological aging. Yet, the metrologic properties of TMS-related measurements is still limited in the aging population.

**Objectives:** The aim of this cross-sectional study was to document the reliability and smallest detectable change of TMS measurements among community-dwelling seniors. A secondary objective was to test if TMS measurements differ between elders based on lifestyle, medical and socio-demographic factors.

**Methods:** Motor evoked potentials (MEPs) elicited by single-pulse TMS were recorded in the first dorsal interosseous (FDI) in 26 elderly individuals (mean age = 70 ± 3.8 years). Resting motor threshold (rMT), MEP amplitudes and contralateral silent period (cSP) were measured on two separate occasions (1-week interval), and the standard error of the measurement (SEM_eas_), intraclass correlation coefficient (ICC), and smallest detectable change in an individual (SDC_indv_) were calculated. Lifestyle, medical and socio-demographic factors were collected using questionnaires. TMS-related outcomes were compared using independent sample *t*-test based on the presence of chronic health diseases, chronic medication intake, obesity, history of smoking, physical activity levels, gender, and level of education.

**Results:** rMT and cSP measures were the most reliable outcomes, with the lowest SEM_eas_ and highest ICCs, whereas MEP amplitude-related measures were less reliable. SDC_indv_ levels were generally high, even for rMT (7.29 %MSO) and cSP (43.16–50.84 ms) measures. Although not systematically significant, results pointed toward a higher corticospinal excitability in elderly individuals who were regularly active, who had no chronic medical conditions and who did not take any medication.

**Conclusion:** Even though SDC_indv_ levels were relatively high, these results show that rMT and cSP are the most reliable outcomes to investigate age-related changes in the corticomotor system and suggest that the influence of factors such as lifestyle habits and medications on TMS measures should be investigated further.

## Introduction

Normal aging is characterized by a decline in cognitive and sensorimotor functions, paralleled by progressive changes in the CNS structure, function, and biochemistry (for reviews cf. Seidler et al., 2010; Turner and Spreng, 2012; Rossini et al., 2015). The neurophysiological mechanisms underpinning age-related decline in motor performance can be probed by TMS (reviewed in Levin et al., 2014). TMS is a safe and non-invasive method to assess the excitability and integrity of the motor cortex and the corticospinal tract, as well as intracortical and interhemispheric inhibitory/excitatory mechanisms involved in motor control (Kobayashi and Pascual-Leone, 2003; Reis et al., 2008; Rossi et al., 2009; Rossini et al., 2015). Since its introduction by Barker et al. (1985), TMS has been increasingly used worldwide, and its basic principles and guideline procedures are described in reference publications (e.g., Groppa et al., 2012; Rossini et al., 2015). In short, a time-varying magnetic field is induced by an electrical current passing through a magnetic coil. When the coil is placed over the primary motor cortex (M1) on the subject’s scalp, the magnetic pulse trans-synaptically depolarizes pyramidal neurons in M1 via cortical horizontal connections. With sufficient stimulus intensity, descending volleys are elicited in the corticospinal tract and a MEP is recorded in contralateral muscles with surface EMG electrodes. Several outcomes can be derived from MEP recordings based on various single and paired-pulse paradigms, with the targeted muscle at rest or slightly contracted (Groppa et al., 2012; Rossini et al., 2015).

Previous TMS studies which have compared young and older adults provided evidence of altered M1 excitability and inhibitory/excitatory mechanisms with age, which were sometimes directly correlated to sensorimotor deficits (Levin et al., 2014). For example, two studies from Fujiyama’s group (Fujiyama et al., 2009, 2012) revealed that younger adults exhibited longer cSP [i.e., a transient suppression of the ongoing EMG activity caused inhibitory mechanisms (Paulus et al., 2008; Rossini et al., 2015)] compared to older participants. Interestingly, cSP length was linked to motor performance in the older group executing more difficult inter-limb coordination tasks; those having the lowest performance showed the shortest cSP durations (Fujiyama et al., 2012). Other research groups reported lower M1 and corticospinal excitability in old vs. young adults (Rossini et al., 1992; Pitcher et al., 2003; Oliviero et al., 2006), as revealed by higher motor thresholds (i.e., the lowest TMS intensity required to elicit a MEP), lower MEP amplitudes and altered properties of the input–output recruitment curves (RC, representing the relation between a wide range of TMS intensities and the resulting MEP amplitudes – for more details cf. Groppa et al., 2012; Rossini et al., 2015).

However, the psychometric evaluation of TMS-related measurements (i.e., validity, reliability, responsiveness, cf. Portney and Watkins, 2009) is still limited (Schambra et al., 2015). Evidence pointed out so far that most TMS outcome measures present with a certain degree of variability caused by various biological and methodological factors (Kiers et al., 1993; Schulz and Ferbert, 1996; van der Kamp et al., 1996; Maeda et al., 2002; Wassermann, 2002; Orth et al., 2003; Orth and Rothwell, 2004; Darling et al., 2006; Rosler et al., 2008; Jung et al., 2010; Roberts et al., 2011). Such variability put into question the reliability of TMS measures, i.e., their accuracy and consistency in stable individuals (Portney and Watkins, 2009). To date, the reliability of TMS measures has been mostly studied in healthy adults (cf. systematic review by Beaulieu et al., 2017) and stroke populations (Koski et al., 2007; Liu and Au-Yeung, 2014; Schambra et al., 2015), but results are still scarce and extremely variable across studies and only a few studies investigated the geriatric population (Wolf et al., 2004; Christie et al., 2007; Schambra et al., 2015). This is problematic since previous studies that focused on the impact of age on TMS measurements showed that TMS outcomes differ between younger and older adults (Pitcher et al., 2003; Smith et al., 2011), hence limiting the generalization of most TMS-reliability evidence to the elderly. More research investigating the reliability of TMS measures in the elderly population could thus improve our collective understanding and appropriate use of this neurophysiological tool for studying how motor control is affected in the aging brain.

Interestingly, a recent literature review pointed out that results are sometimes controversial between papers that investigated age-related changes with TMS (Levin et al., 2014). Based on the heterogeneity that characterizes the elderly population, it can be hypothesized that factors such as lifestyle habits and socio-demographic characteristics could in part explain the variable results in the TMS literature. Cumulative neuroimaging evidence already support that these factors might have a strong impact on the CNS (Mahncke et al., 2006; Vaynman and Gomez-Pinilla, 2006; Seidler et al., 2010; Debette et al., 2011; Habes et al., 2016), but an investigation tackling how such factors can influence age-related changes in the brain has never been attempted with TMS.

The primary aim of this cross-sectional study was to characterize the absolute reliability (a.k.a. measurement error), relative reliability and smallest detectable change levels for an individual of TMS-related measures within the elderly population. Based on the results obtained in the literature (Badawy et al., 2011; McGregor et al., 2012; Liu and Au-Yeung, 2014; Schambra et al., 2015; Beaulieu et al., 2017), we hypothesized that the rMT and the cSP would be the most reliable outcome, conversely to all MEP amplitude-related outcomes. As a secondary objective, we performed exploratory analysis to compare TMS-related outcomes based on the presence of chronic health diseases, chronic medication intake, obesity, history of smoking, physical activity levels, gender, and level of education among functionally independent and community-dwelling seniors without any known cognitive or neurological disorders. Based on the literature, we hypothesized that participants having poorer health and lifestyle habits (sedentarity, history of smoking, obesity, chronic medication intake, and chronic diseases) would exhibit shorter cSP durations and lower M1 and corticospinal excitability (characterized by higher motor thresholds, lower MEP amplitudes and lower slopes of input–output RC).

## Materials and Methods

### Participants

Twenty-six elderly individuals were recruited in the general population of the Sherbrooke area after providing informed written consent approved by the local ethics committee. The participants’ characteristics are detailed in Table 1. The inclusion criteria were: age ≥ 65 years old; functionally independent in their activities of daily living; good vision. The general exclusion criteria were: any cognitive, psychiatric, or neurological disorders; any upper limb orthopedic injury or disease. Specific exclusion criteria related to TMS further included a history of seizures, cardiac pacemaker and intracranial metallic implants (Rossi et al., 2009). Participants were instructed not to change their medication intake and other lifestyle habits during the study.

**Table 1:** Characteristics of participants.

General and socio-demographic characteristics	
Participants (N)	26
Age (mean ± SD)	70.0 ± 3.8 years
Gender (N: males/females)	13/13
Handedness (N: right/left)	26/0
Race	
- Caucasian/other	26/0
Highest education level (N)	
- Secondary	11
- Post-secondary	15
Anthropometry (mean ± SD)	
- Height	1.64 ± 0.11 m
- Weight	72.4 ± 13.8 kg
- BMI	26.7 ± 2.9 kg/m^2^
- Overweight^∗^ (N: yes/no)	19/7
**Lifestyle habits**	
Physical activity^∗∗^ (N: regular/sedentary)	19/7
Smoking (N: yes/no)	
- Currently smoking	0/26
- History of smoking	13/13
**Medical characteristics**	
Chronic disease (N: yes/no) - Diagnostic (N)	15/11-Arthritis (3); Benign prostatic hyperplasia (2); Crohn disease (1); Diabetes (2); Hypercholesterolemia (5); Hypertension (10); Hypothyroidism (3); Intercostal neuralgia (1); Osteoarthritis (4); Sleep apnea (1); Tinnitus (1)
Chronic medication intake (N: yes/no)	17/9
- Main effect (Drug)^∗∗∗^	Anticholesteremic (Atorvastatin, Rosuvastatin, **Simvastatin**); Anticoagulant (**Rivaroxaban**); Antidepressant/anxiolytic (**Escitalopram**, **Hydroxyzine**, **Trazodone**, **Venlafaxine**); Antidiabetic (Glyburide, **Metformin**, **Pioglitazone**, **Saxagliptin**, **Sitagliptin**); Antihypertensive (Amlodipine, **Amiloride**, Cilazapril, **Diltiazem**, Hydrochlorothiazide, **Irbesartan**, Lisinopril, Losartan, Metoprolol, Quinapril, **Telmisartan**, Valsartan); Anti-inflammatory (**Acetaminophen**, **Acetylsalicylic acid**, **Celecoxib**, Hydroxychloroquine); Antiosteoporotic (**Alendronate**); BPH-medication (**Alfuzosin**, **Dutasteride**, **Tamsulosin**); Chemotherapy agent/immune system suppressant (Methotrexate); Gastric acid inhibitor (Esomeprazole, Omeprazole, Pantoprazole); Hormone replacement therapy (**Estrone**, Levothyroxine, **Premarin**); Laxative (Docusate); Topical corticosteriod (**Clobetasol**); Uric acid synthesis inhibitor (**Allopurinol**)


*^∗^Overweight is defined as BMI ≥ 25. ^∗∗^Regular physical activity was defined as ≥ 1 session per week. ^∗∗∗^Drugs in bold are those passing through the blood brain barrier (source: www.drugbank.ca). BMI, body mass index (weight/height^2^); N, number of participants.*

### Experimental Procedure

The study took place at the Research Centre on Aging (Sherbrooke, QC, Canada) and consisted in two visits separated by a 1-week interval. TMS procedures were strictly replicated between the two testing sessions by the same investigator per participant. All relevant participant-related information, consisting of lifestyle habits (frequency of physical activity, smoking), medical (chronic diseases and chronic medication intake) and socio-demographic (age, height, weight, race, highest completed level of education) factors were collected using a questionnaire.

### Electromyography (EMG)

After standard skin preparation (Hermens et al., 2000), two Ag/AgCl surface recording electrodes (1 cm^2^ recording area) were positioned following a belly-tendon montage on the FDI muscle of the dominant hand. More specifically, one electrode was placed over the belly muscle and the other was fixed on the lateral side of the second metacarpo-phalangeal joint. A third ground electrode was secured over the radial styloid process. The EMG signals were amplified and filtered (200 Hz – 2 kHz) with a CED 1902 amplifier (Cambridge Electronic Design Limited, Cambridge, United Kingdom) and digitized at a sampling rate of 10 kHz with a Power 1401 mk II interface and Spike 2 software (version 7.10; Cambridge Electronic Design Limited, Cambridge, United Kingdom). All TMS outcomes required complete muscle relaxation (except for cSP), EMG signals prior to each TMS impulse were visually monitored online and inspected during *post hoc* analyses. Any trial in which pre-impulse electrical activity was detected was discarded.

### Transcranial Magnetic Stimulation (TMS) Procedures

Participants were comfortably seated on a standard chair throughout TMS testing procedures, which respected published guidelines in the field (cf. Groppa et al., 2012; Rossini et al., 2015). Magnetic stimuli (monophasic waveform) were delivered using a 70 mm figure eight coil connected to a Magstim 200 TMS device (Magstim Company, Ltd., United Kingdom). The coil handle was held approximately perpendicular to the skull and oriented at 45° in the mid-sagittal plane to induce a posterior-to-anterior electrical current direction in the cortex. The optimal site for eliciting MEPs in the contralateral FDI with the lowest stimulation intensity (hotspot) was first determined by moving the coil 1 cm at a time over the hand’s M1 area [located approximately 1 cm anterior and 4 cm lateral to the vertex based on the 10–20 EEG system (Klem et al., 1999)] while stimulating at 40% of the stimulator’s maximum output (MSO). The TMS intensity was increased above 40% MSO if no MEP was elicited. Once located, the coil’s position above the hotspot was traced on a bathing cap worn by the subject, and the experimenter regularly verified that the coil was correctly positioned throughout the session. These procedures were precisely replicated on the second visit.

#### Resting Motor Threshold (rMT)

The rMT was defined as the lowest TMS intensity (in %MSO) required to induce MEPs amplitude of over 50 μV in four out of seven trials in the resting FDI. To this end, TMS intensity was slowly decreased by 2% steps until MEPs ≥ 50 μV were no longer elicited. The intensity was then increased by 1% steps until the rMT criterion was met.

#### Recruitment Curve (RC)

At rest, five blocks of different stimulation intensity (i.e., 90, 110, 120, 130, and 150% of the rMT) were randomly provided to participants, each block consisting in 10 magnetic stimuli. Time between each magnetic stimulus was randomly varied between 5 and 10 s, by the computer. MEPs that were below 50 μV were excluded from the analysis (except at 90% of rMT). Recruitment curves were constructed by expressing the mean peak-to-peak MEP amplitudes (y-axis) based on the corresponding stimulus intensity in % rMT (x-axis).

#### Contralateral Silent Period (cSP)

Contralateral silent periods were obtained from two stimulation blocks at 120 and 130% of the rMT (in %MSO), each block consisting of 10 TMS stimuli separated by 5–10 s (randomly determined by the computer). TMS stimulations were delivered while the subjects sustained a slight isometric activation with their FDI (thumb-index pinch) at 10% of MVC, determined by the average of three trials in which participants were asked to apply their maximum pressure on a pinch gauge (North Coast Medical, Inc., Morgan Hill, CA, United States). During the trials, participants were asked to maintain 10% of their MVC, which was monitored visually by the evaluator on the pinch gauge.

### Data Pre-processing and Statistical Analysis

Motor evoked potential amplitudes and cSP durations were measured offline with the Spike 2 software. Precisely, a script automatically marked the exact time at which the TMS stimulation was triggered and then calculated the MEP peak-to-peak amplitude within a 100 ms window starting 10 ms post-stimulation. The cSP *onset* (start of the cSP) was defined as the time where the EMG signal returned to the baseline (i.e., no EMG activity) after the MEP and the cSP *offset* (end of the cSP) was defined as the return of the voluntary contraction. These points were determined visually from the raw EMG recordings. Concerning RC outcomes, the expression of MEP amplitude in function of TMS intensity habitually results in an “s-shaped” curve that can be best fitted using the Boltzmann sigmoidal function (Devanne et al., 1997), providing several measures having interpretable neurophysiological underpinnings (e.g., MEP amplitude plateau, slope of the Boltzmann curve, etc., cf. Devanne et al., 1997). However, in the present study, RC data with the older population rather resulted in a relatively linear increase of MEPs amplitude [a situation which was observed by other researchers – see for instance (Chen et al., 1998)]. Precisely, a sigmoidal fit was only realizable in six out of the 26 participants. Hence, the Boltzmann equation was impossible to compute, and mean slopes were obtained by using linear regression analyses.

Statistical analyses were performed using the Statistical Package for the Social Sciences (SPSS) software (version 17). Statistical assumptions for parametric tests and reliability analyses were explored by determining the data’s normality and homoscedasticity (Atkinson and Nevill, 1998; Weir, 2005; Portney and Watkins, 2009). Normality was assessed by visual screening of histogram distributions and by the Shapiro–Wilk test allowing us to assume that the data were normally distributed (Henderson, 2006; Ghasemi and Zahediasl, 2012). Homoscedasticity [i.e., the absence of heteroscedasticity (Weir, 2005)] was tested following the procedure detailed in Damron et al. (2008), that is by exploring the correlation between the size of the measurement error and the magnitude of the observed scores. For example, in the presence of heteroscedasticity, individuals with higher scores would show a higher random error measure than those with lower scores. As recommended, whenever non-normal distributions or heteroscedasticity were found, a logarithmic (natural) transformation of the outcome was applied (Atkinson and Nevill, 1998; Schambra et al., 2015; Beaulieu et al., 2017), and further analyses were performed on the log-transformed data.

For the study’s primary objective, the measurement error of TMS-related outcomes was evaluated using the standard error of the measurement (SEM_eas_) (Weir, 2005). SEM_eas_ is expressed in the same unit as the measure; the smaller its value, the more reliable the measure is Weir (2005). The following formula was used: SEM_eas_ = √MSE where MSE is the mean squared error term (or “residual error”) obtained from the ANOVA applied on test and retest measurements (Weir, 2005). Each SEM_eas_ was normalized to the pooled mean from both testing sessions (named SEM_eas_%), using the formula SEM_eas_% = SEM_eas_/pooled mean^∗^100. This method enabled the comparison of the measurement error between TMS outcomes by expressing them on the same scale (%) (Lexell and Downham, 2005; Schambra et al., 2015). We used the cut-off of <10% as an arbitrary value for suggesting low measurement error (Schambra et al., 2015). The smallest detectable change for an individual or SDC_indv_ (a.k.a. minimal detectable change or smallest real difference) was calculated from SEM_eas_ scores, as follows: SDC_indv_ = SEM_eas_^∗^1.96^∗^√2 (Beckerman et al., 2001). Relative reliability (a.k.a. reliability_MP_, cf. Mokkink et al., 2010) was tested by the ICC (Weir, 2005). Our study considered ICC scores to be good from 0.75 to 0.89 and excellent if ≥ 0.90, although no absolute benchmarks exist for the interpretation of ICC scores (Weir, 2005; Portney and Watkins, 2009). ICCs of TMS measures were calculated using the two-way random effects “absolute agreement” model in SPSS (a.k.a. model 2,k – cf. Weir, 2005). The presence of systematic differences in TMS measures between sessions 1 and 2 was explored using paired sample *t*-tests.

For the secondary objective, subgroups depending on behavioral, medical, and socio-demographic factors were created, based on the following dichotomous classifications: male/female; presence/absence of a diagnosed chronic disease, independently of the type of disease; presence/absence of chronic medication intake; highest level of education secondary (a.k.a high school/post-secondary); regular physical activity ≥ 1/ < 1 session per week; history of smoking/never smoked. Independent sample *t*-tests were used to compare all TMS outcomes between the subgroups. Statistical significance was set at *p* < 0.05 for all analyses.

## Results

### Participants’ Characteristics

The general, socio-demographic, lifestyle habits and medical characteristics of the 26 participants are detailed in Table 1. All were right-handed (mean age = 70.0 ± 3.8 years). Most second objective subgroups were balanced, particularly gender (13 males and 13 females), history of smoking (13 smoked in the past and 13 did not), highest level of education (15 post-secondary and 11 secondary) and chronic disease (15 having one or more chronic disease and 11 having none). The other subgroups consisted in chronic medication intake (17 taking one or more medication on a regular basis and 9 taking none), BMI (19 being considered overweight and 7 were not) and physical activity (19 were regularly active and 7 were not). Most chronic illnesses consisted in arthritis/osteoarthritis and vascular risk factors (diabetes, hypertension, hypercholesterolemia), conditions which are highly prevalent in the elderly population (Espeland et al., 2017). Furthermore, 13 participants took at least one drug known to pass through the blood brain barrier (these drugs are in bold in the Table 1), and five subjects were prescribed antidepressant/anxiolytic medications, potentially influencing brain activity via the inhibition of serotonin/norepinephrine reuptake mechanisms (Paulus et al., 2008; Ziemann et al., 2015). Of note, the subgroups “chronic disease” and “medication intake” were comprised of the same individuals, except for two women who were taking estrogen replacement therapy, as menopausal symptoms were not considered as a chronic disease.

### Missing TMS Data

Two participants included in this study had exceptionally high rMT (%MSO) values, which made it impossible to record MEP values at 150% rMT (*n* = 2) and 130% rMT (*n* = 1) since it exceeded the capacity of the stimulator (i.e., over 100% MSO). Less than 5% of all the MEPs obtained at suprathreshold intensity were below the 50 uV threshold and were excluded from the analysis.

### Raw TMS Outcomes

Raw (i.e., untransformed) TMS measures are presented in Table 2 for the entire group. Between-session comparisons revealed no systematic difference in TMS outcome measures, except for rMT which showed a slight increase of 1.8 ± 3.7 %MSO (*p* = 0.02) between sessions 1 and 2. MEP amplitudes and RC mean slope data showed non-normal distributions or heteroscedasticity. Following natural log transformation, MEP amplitudes and RC mean slope data was normally distributed and the assumption of homoscedasticity was respected. All further analyses for MEP amplitudes and RC mean slope were therefore realized on log-transformed data.

**Table 2:** Raw TMS measures.

	Session 1	Session 2
	*Mean ± SD (CV)*	*Mean ± SD (CV)*
rMT (%MSO)	49 ± 12 (24.5)	51 ± 12 (23.5)^∗^
MEP amplitude (μV)		
- 90% rMT	21.58 ± 11.66 (54.0)	23.57 ± 16.10 (68.3)
- 110% rMT	216.01 ± 194.04 (89.8)	232.08 ± 173.53 (74.8)
- 120% rMT	269.33 ± 171.01 (63.5)	318.52 ± 206.01 (64.7)
- 130% rMT	372.00 ± 196.10 (52.7)	434.32 ± 337.07 (77.6)
- 150% rMT	477.97 ± 266.38 (55.7)	552.94 ± 444.05 (80.3)
RC mean slope (μV/% rMT)	7.52 ± 4.31 (57.3)	8.50 ± 7.65 (90.0)
cSP duration (ms)		
- 120% rMT	92.89 ± 36.77 (39.6)	95.09 ± 31.13 (32.7)
- 130% rMT	97.37 ± 44.86 (46.1)	99.74 ± 43.19 (43.3)


*^∗^Significant difference (*p* < 0.05) between sessions. Underlined TMS outcomes were those that required a natural log transformation because of non-normal distributions or heteroscedasticity. cSP, contralateral silent period; CV, coefficient of variation [100^∗^(SD/mean)]; MEP, motor evoked potentials; MSO, maximal stimulator output; RC, recruitment curve; rMT, resting motor threshold.*

### Reliability

Results for measurement error, smallest detectable change for an individual (SDC_indv_) and reliability_MP_ are presented in Table 3, along with the pooled means and CVs calculated from the two sessions that are critical for having a correct interpretation and generalization of ICC results (Schambra et al., 2015; Beaulieu et al., 2017). Indeed, high data dispersion (i.e., high CVs) increases the chance of obtaining high ICC scores (Weir, 2005; Schambra et al., 2015). Of note, reliability results for MEP amplitudes and RC mean slope are given in both log transformed and raw data (light gray in Table 3). Although log transformed results are more valid because they were corrected for non-normal or heteroscedastic distributions), we have decided to also present the reliability of untransformed measures to ease the interpretation of the results.

**Table 3:** Reliability results (*n* = 26).

	Measurement error			Reliability			Pooled data	
			
*TMS outcomes (units)*	SEM_eas_	%SEM_eas_	SDC_indv_	ICC	Lower 95%CI	Upper 95%CI	Mean	CV
rMT (%MSO)	2.63	5.30	7.29	0.970	0.922	0.987	49.67	23.76
LogN_MEP amplitude (NA)								
- 110% rMT	0.51	NA	1.42	0.468	0	0.763	NA	NA
- 120% rMT	0.45	NA	1.24	0.542	0	0.794	NA	NA
- 130% rMT	0.51	NA	1.42	0.511	0	0.787	NA	NA
- 150% rMT	0.53	NA	1.46	0.472	0	0.774	NA	NA
*Raw MEP amplitude (μV)*								
*- 110% rMT*	*158.99*	*70.97*	*440.70*	*0.413*	*0*	*0.740*	*224.04*	*81.43*
*- 120% rMT*	*152.60*	*51.91*	*422.98*	*0.516*	*0*	*0.781*	*293.92*	*64.35*
*- 130% rMT*	*234.61*	*58.19*	*650.32*	*0.434*	*0*	*0.750*	*403.16*	*68.14*
*- 150% rMT*	*335.90*	*65.17*	*931.06*	*0.277*	*0*	*0.689*	*515.45*	*70.66*
LogN_RC mean slope (NA)	0.55	NA	1.53	0.517	0	0.785	NA	NA
*Raw RC mean slope (μV/%rMT)*	*5.53*	*69.11*	*15.34*	*0.346*	*0*	*0.709*	*8.01*	*77.00*
cSP duration (ms)								
- 120% rMT	15.57	16.56	43.16	0.886	0.747	0.949	93.99	35.91
- 130% rMT	18.34	17.87	50.84	0.885	0.733	0.950	102.66	38.36


*Results in light gray color are those obtained from untransformed measured (even though they showed non-normal or heteroscedastic distributions). 95%CI: 95% confidence intervals; cSP, contralateral silent period; CV, coefficient of variation; ICC, intraclass correlation coefficient; LogN, data transformed using a natural logarithm; MEPs, motor evoke potentials; MSO, maximal stimulator output; NA, not applicable (for log transformed outcomes); RC, recruitment curve; rMT, resting motor threshold; SDC, smallest detectable change; SEM_*eas*_, standard error of the measurement.*

Measurement errors were low for rMT (%SEM_eas_: 5.30%), high for all MEP amplitudes and RC mean slope (51.91–70.97%) and moderate for cSP measures (16.56–17.70%). ICCs provided evidence of excellent reliability for rMT and cSP, which were particularly high for the former with an ICC and 95% confidence intervals above 0.90 (see Table 3), even though pooled CVs were not particularly high (23.78–38.36%). Inversely, all remaining outcomes (MEP amplitude and RC mean slope) showed low ICCs with very large confidence intervals, despite important data dispersion (all pooled CVs above 60%; see Table 3).

SDC_indv_ were relatively high for all TMS measures. For instance, in order to conclude with 95% confidence that a change occurred when measuring the rMT with the elderly population, an increase/decrease would need to reach more than 7.29 %MSO (see Table 3 for SDC_indv_ levels of all TMS outcomes).

### Comparisons Between Subgroups

#### General and Socio-Demographic Characteristics

Compared to men, women were significantly lighter (*p* < 0.001) and smaller (*p* < 0.001), but the BMI (in kg/m^2^) was not different (*p* = 0.37). No difference in TMS measures was found between subgroups, based on socio-demographic characteristics (i.e., gender, education level, and BMI; all *p*-values > 0.17).

#### Lifestyle Habits

**FIGURE 1:**
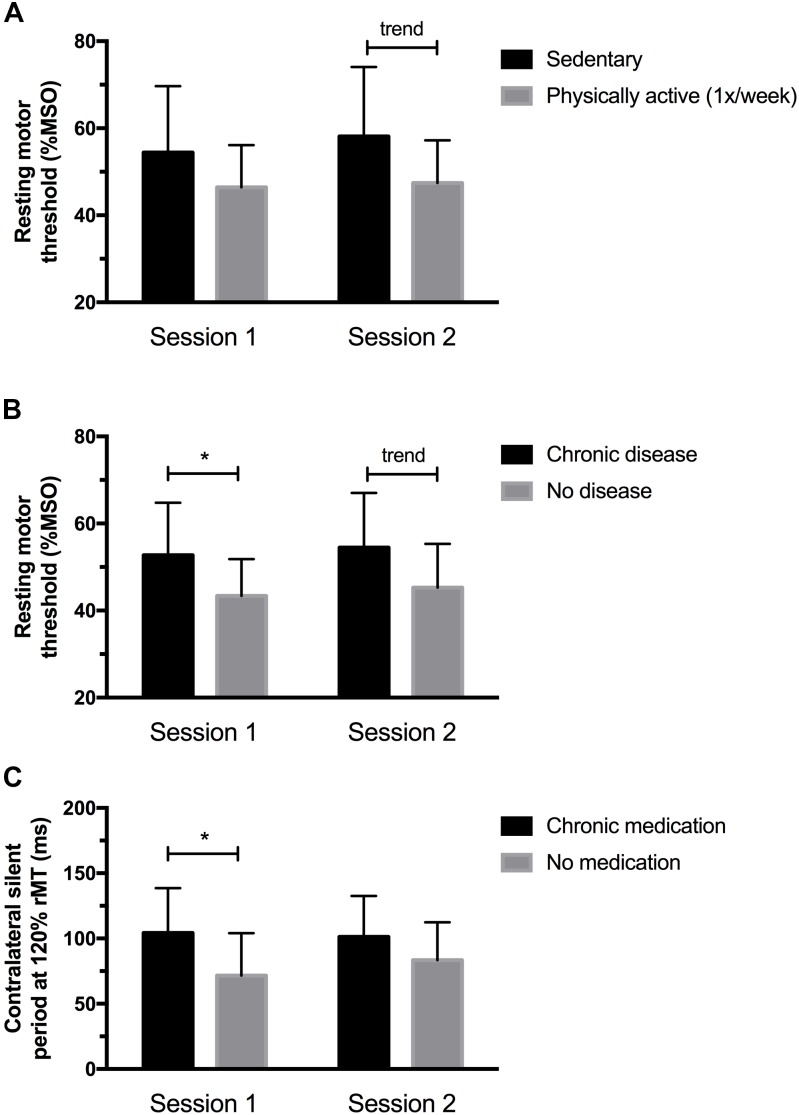
**(A)** Physically active individuals had lower rMT at session 2 but not at session 1. **(B)** Individuals having chronic medical conditions showed higher rMT values at session 1, and a significant trend (*p* = 0.06) was observed at session 2. **(C)** Participants taking medication showed longer cSP at 120% rMT at session 1; no differences in cSP were noted at session 2. ^∗^Significantly different (*p* < 0.05) between sessions; trend (*p* < 0.10).

As shown in Figure 1A, physically active individuals (i.e., ≥1 session of physical activity per week) had lower rMT at session 2 (*p* = 0.05), but not at session 1 (*p* = 0.13). The difference between subgroups were 7.7 %MSO ( ± 4.95) at session 1 and 10.4 %MSO ( ± 5.11) at session 2. No other significant difference based on behavioral characteristics was found for the remaining TMS outcomes (all *p*-values > 0.1).

#### Medical Characteristics

**FIGURE 2:**
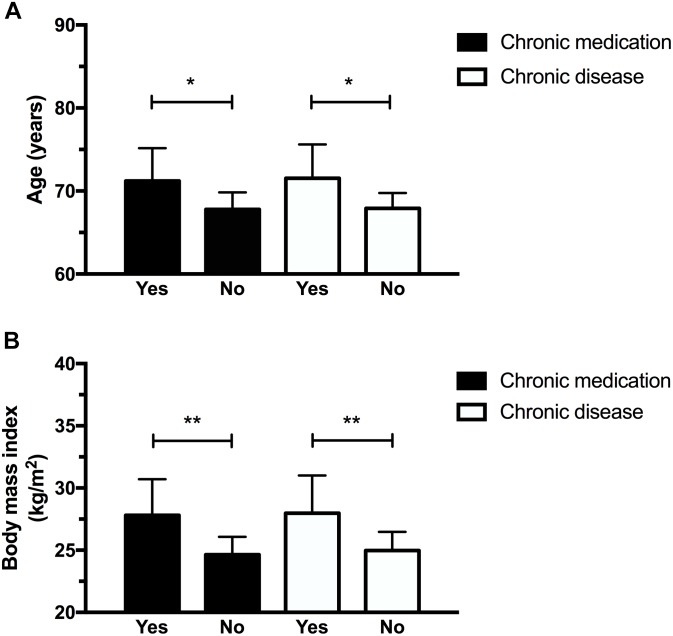
Participants having chronic diseases and who took medication were older **(A)** and had higher body mass index **(B)**. ^∗^*p* < 0.05; ^∗∗^*p* < 0.01.

Figures 1 and 2 illustrate the differences observed between subgroups based on their medical characteristics. First, individuals having chronic medical conditions showed higher rMT values at session 1 (*p* = 0.04, mean difference: 9.4 ± 4.25 %MSO) which was nearly significant at session 2 (*p* = 0.06, mean difference: 9.2 ± 4.59 %MSO; see Figure 1B). These effects were not found for the subgroup taking medication on a regular basis (*p*-values > 0.1; mean difference observed at session 1 = 6.4 ± 4.7 %MSO; mean difference observed at session 2 = 6.3 ± 5.0%). cSP at 120% rMT were longer in the medicated subgroup at session 1 (*p* = 0.03; mean difference: 32.7 ± 13.95 ms), suggesting increased intracortical inhibition in individuals who took medication. However, no differences in cSP were noted at session 2 (*p* = 0.17, mean difference 17.88 ± 12.58 ms; see Figure 1C). Of note, those suffering from chronic illnesses and taking medications on a regular basis were older and had a higher BMI than “healthy” subgroups (see Figures 2A,B). Precisely, compared to their counterparts, the “chronic disease” and “medication intake” subgroups respectively showed mean differences of 3.4 ± 1.4 years (*p* = 0.01) and 3.6 ± 1.3 years (*p* = 0.03), 3.0 ± 1.0 kg/m^2^ (*p* = 0.01) and 3.15 ± 1.03 kg/m^2^ (*p* = 0.01). To further explore the potential influence of age and BMI level on rMT and cSP results, Pearson’s correlation analyses were conducted for the whole group, and for each subgroup (disease and medication) at each session. The analyses revealed no relationship between rMT/cSP and age and between rMT/cSP and BMI (all *p*-values > 0.1).

## Discussion

### Main Findings

The present study assessed the measurement error, smallest detectable changes, and relative reliability of TMS-related measurements among functionally independent and community-dwelling seniors. Along with our previous hypothesis, the rMT and cSP were the most reliable TMS-outcomes in the elderly while the MEP amplitude-related outcomes (MEP amplitude and RC) had lower reliability.

This study also compared TMS outcome measures in community-dwelling elders based on socio-demographic, lifestyle habits and medical factors. To our knowledge, this is the first study investigating these questions, as previous work in the field tended to focus on the “age” factor only. Although a few limitations might affect the strength of our findings, results mostly supported our initial hypotheses, as corticospinal excitability was lower in elders with poorer lifestyle habits/general health when compared to those presenting with healthier behaviors/fewer medical problems.

### Reliability and Smallest Detectable Change of TMS Measures in the Elderly

Our reliability results showed a lower percentage of error and higher ICCs for the rMT and cSP compared to the other TMS measures, which is in line with the literature (Badawy et al., 2011; McGregor et al., 2012; Liu and Au-Yeung, 2014; Schambra et al., 2015; Beaulieu et al., 2017). These results further support the use of these TMS measures for both evaluative and diagnostic/prognostic purposes with the geriatric population (Weir, 2005; Schambra et al., 2015; Beaulieu et al., 2017). Future studies with similar population characteristics will be able to use the reliability metrics provided in Table 3, for example to anticipate how much an individual (SDC_indv_) or group [SDC_indv_ divided by the square root of their sample size (see Terwee et al., 2007; Schambra et al., 2015)] would have to change to be above the measurement’s error. This provides an analytic method that is complementary to standard hypothesis testing (Schambra et al., 2015). For example, although differences in rMT between subgroups based on lifestyle habits were not always statistically significant in this study, they were above the group SDC levels.

Conversely to the rMT and cSP, MEP amplitudes and RC mean slope presented with relatively high SEM_eas_ and SDC_indv_ levels, and with low ICC scores. Many previous reports also obtained poor reliability metrics in younger populations, particularly for MEP amplitudes (Livingston and Ingersoll, 2008; Fleming et al., 2012; Ngomo et al., 2012; Beaulieu et al., 2017). Our results might be due in part to our inability to fit the Boltzmann function (Devanne et al., 1997) in most participants; future studies are thus encouraged to collect supplemental TMS intensities to construct a reliable RC sigmoidal curve with the older population, particularly at higher TMS intensities to ensure that a MEP plateau is reached. Clearly, the very high measurement errors and low ICCs (observed even after having performed a log natural transformation), call for a change of practice in MEP amplitude acquisition. Recent studies looked into the effect of the number of delivered stimuli (cf. reviews by Beaulieu et al., 2017; Cavaleri et al., 2017), or the use of electroencephalography to control the timing of TMS pulse delivery, depending on the ongoing state of the brain’s oscillatory activity (Ferreri et al., 2014; Keil et al., 2014; Zrenner et al., 2018). In one of these studies, Chang et al. (2016) used Cronbach alpha to document the internal consistency of TMS measures obtained with paired-pulse paradigms. They showed that about 20–25 trials are required to reach an appropriate level of trial-to-trial consistency for paired-pulse outcomes. Although their work brings important knowledge to the field of TMS, internal consistency should not be interpreted the same way as SEMeas, ICC and SDC, as it mostly considers only one source of variability (i.e., intrinsic physiological variability) if the setup and evaluator are kept the same. Other studies tried to determine if the number of stimuli could influence reliability scores (so far, only with the ICC), but results are inconsistent (see the recent review on the topic by Cavaleri et al., 2017). It should also be remembered that ICC is probably not the best indicator, as it does not describe measurement variability/error (Weir, 2005; Schambra et al., 2015; Beaulieu et al., 2017). Recording 20–25 stimuli for TMS measures of intracortical inhibition and facilitation is probably ideal (Ngomo et al., 2012; Beaulieu et al., 2017). However, this practice might not be reasonable to produce RC curves (i.e., 20–25 trials for each TMS intensity), especially in older populations, as this lengthens the procedures and could introduce physical and cognitive fatigue.

Comparing our data with the available evidence in literature is difficult, based on the very high heterogeneity across published studies on TMS reliability (Beaulieu et al., 2017), and because reliability indices such as ICC are not easily generalizable as it depends on the sample dispersion in a way that higher inter-subject variation tends to increase ICC values, and vice-versa (Weir, 2005). Interestingly, the lower reliability scores for RC measures in our study – as compared to papers having tested younger adults (Beaulieu et al., 2017) – encourage future work to compare the reliability between younger and older adults. Having higher/lower variability of TMS measures in elders could lead to further research on the neurophysiological underpinnings of such differences. For instance, it could be hypothesized that brain atrophy with age has a general dampening effect on TMS variables (Rossini et al., 1992; Pitcher et al., 2003; Oliviero et al., 2006; Levin et al., 2014), hence reducing *inter*-subject variation (potentially resulting in lower ICCs) and limiting the ability of physiological processes tested by TMS to vary (potentially resulting in less measurement error). Alternately, an increase in the *intra*-individual variability of cognitive and perceptivo-motor functions has been observed and proposed as a biomarker of aging (MacDonald et al., 2006), hence potentially increasing the within-subject measurement error and resulting in both lower relative (ICC) and absolute reliability (SEM_eas_). Knowing that the intrinsic variability of TMS measures could be in part linked to normal fluctuations of physiological processes (Rossini et al., 2015), it is expected that older persons will exhibit higher trial-to-trial variation, thus affecting both relative and absolute reliability. These important questions should be tackled in future work.

### Comparisons Based on Socio-Demographic, Lifestyle Habits, and Medical Factors

Resting motor thresholds tended to be higher in the presence of chronic diseases, medication intake and sedentary behaviors. Although the results were not particularly strong and not systematically significant in visit 1 and visit 2, these observations point toward a higher excitability of M1 and the corticospinal tract (lower rMT values) in “healthier” subgroups, thus suggesting that these behavioral and medical factors might influence age-related changes within the motor cortex. Evidence from other neurophysiological tools than TMS already highlighted the impact of lifestyle habits and medical affections on the CNS. For instance, a recent large-scale study using magnetic brain imaging with 592 community-dwelling elders observed a significant and positive correlation between the level of brain atrophy and the annual medical expenditure which represents a financial way to evaluate the health status of a person (Last et al., 2017). Moreover, the presence of vascular risk factors and chronic diseases, such as those that were prevalent in our recruited sample, has been shown to increase brain atrophy in elders (Debette et al., 2011; Habes et al., 2016). Other transversal studies observed that physically active elders can recruit additional brain resources to perform cognitive and motor tasks (Hillman et al., 2002) and have higher brain volume (Colcombe et al., 2003) than their sedentary counterparts.

Interestingly, Colcombe et al. (2003) showed that, when healthy but physically inactive older adults were enrolled in a 6-month aerobic fitness program, both gray and white matter volume increased compared to the control group having only performed stretching and toning exercises. Likewise, a recent randomized controlled trial (Best et al., 2015) with 155 older women reported that those engaged in a 52-week resistance training program vs. those that only performed toning and balance interventions demonstrated better improvements in muscle power and executive functions, and reduced cortical atrophy at the 2 year follow-up; an effect that could be attributed to the release of brain growth factors (Dishman et al., 2006; Vaynman and Gomez-Pinilla, 2006). Based on this evidence, we propose that the potentially lower rMTs in “healthier” subgroups might reflect less brain atrophy and/or richer inter-neuronal networks (higher M1 excitability) within the precentral and motor cortex. Future studies should investigate these observations in more detail, for instance by directly measuring the skull-cortex distance at the hotspot using anatomical magnetic resonance imaging (Stokes et al., 2013). It could be hypothesized that if brain atrophy is the main factor explaining our rMT results, then individuals with the highest threshold should also demonstrate the highest skull-cortex distance. The results obtained in the present study will have to be reproduced in a larger cohort before any clear conclusions can be drawn. If future work provides enough evidence to support our findings, TMS outcome measures such as the rMT could be used as a meaningful indicator of changes in M1 function and excitability in the aging population.

Results obtained with the contralateral silent period did not confirm our initial hypothesis. Based on literature, it was anticipated that longer cSP would be found in healthier subgroups, because younger individuals or elders with better motor skills demonstrated longer cSP as well (Fujiyama et al., 2009, 2012). Conversely, we observed longer cSP in participants taking medications at the first visit. TMS measures of cortical inhibition such as the cSP are habitually linked to motor planning, particularly when performing more complex tasks (Reis et al., 2008; Levin et al., 2014). However, there are discrepancies in the literature about how these intracortical inhibitory/excitatory mechanisms are affected by aging (cf. review by Levin et al., 2014). We propose that part of this controversy might result from unexplored factors, including those investigated in the present work. For instance, five participants took antidepressive/anxiolytic drugs, which can lengthen the cSP via serotonin-specific or serotonin/norepinephrine reuptake inhibitors (Robol et al., 2004), although this effect was not reported in other papers (cf. review by Paulus et al., 2008). However, cSP length is directly influenced by the intensity of the stimulation (Inghilleri et al., 1993; Orth and Rothwell, 2004), and when the intensity is expressed in % rMT as in the present work, longer cSP are more likely to be observed in individuals with a higher rMT (Kimiskidis et al., 2005). Therefore, the shorter cSP obtained in our healthier subgroup might simply reflect the lowest rMT levels. This explanation fits well with the lack of significant difference of cSP length at the second visit, as the difference in rMT levels was also lower then. The use of more comprehensive cSP evaluations, such as finding the cSP threshold or constructing RC curves for cSP lengths (Kimiskidis et al., 2005), should be considered in the future.

### Limitations

Results obtained in the present work might be affected by a few limitations that are worth mentioning. First, a slight but significant increase in rMT was detected between sessions (2%), pointing out the possibility of a systematic error. The participants’ experience of TMS after the first visit might have influenced their attention or level of arousal during the second session, which is recognized as potentially affecting TMS outcomes (Chipchase et al., 2012). Increasing the number of trials for rMT determination could have perhaps helped attenuate these intersession differences. Since rMT determination requires a lot of stimuli before testing the 50% rule of the relative frequency method, we decided to test 4 out of 7 instead of 5 out of 10 to minimize fatigue and reduce experimentation time (see also Groppa et al., 2012 for similar approach). From a metrological perspective, the mean rMT increase of 2% MSO observed in our study was within the measurement error (i.e., 2.63% MSO), and was not systematically observed across individuals, with five participants showing slight decreases of rMT, and three subjects having unchanged rMT. Altogether, these points highlight that our rMT estimation criterion might have introduced a supplemental source of error, and that further research is required to better characterize the strengths and limits of existing rMT procedures (Awiszus, 2014).

It should also be pointed out that sample size was relatively small, and that subgroups were not always balanced for the analyses pertaining to the secondary objective. A small sample size increases the confidence intervals associated with reliability indicators and increases the risk of committing type II errors (Portney and Watkins, 2009). The small number of participants hence challenges the non-significant results regarding our secondary objective, for example regarding the impact of smoking on TMS-related measurements. Future studies looking at the effect of smoking on TMS outcomes should also focus on current smokers vs. individuals who never smoked, a comparison that we were unable to make, as no current smoker was recruited.

## Conclusion

Our findings support the reliable use of rMT and cSP with the aging population and provide reference levels for the smallest detectable change and ICC values that can be generalized to a future use for these TMS measurements in this population. Ultimately, the use of TMS measures could provide meaningful information on age-related changes in the motor cortex and might even be able to predict the development of sensorimotor disorders or functional limitations in the future. This work also provides the first evidence that lifestyle habits and medical conditions may have a significant impact on TMS outcomes in community-dwelling elders. Researchers in the fields of TMS and aging are thus encouraged to consider the potential influence of these factors, especially when “healthy” elders are recruited to provide comparative data for other populations with pathological affections. In the meantime, our results underscore the impact that poor lifestyle habits and chronic diseases might have on the corticospinal system.

## Ethics Statement

This study was carried out in accordance with the recommendations of the ethical standards of the Ethics committee on human research of the Research Center on Aging of the CIUSSS Estrie – CHUS (Project No. 2014-400). The protocol was approved by the human research of the Research Center on Aging of the CIUSSS Estrie – CHUS. All subjects gave written informed consent in accordance with the Declaration of Helsinki.

## Data Availability

The raw data supporting the conclusions of this manuscript will be made available by the authors without undue reservation.

## Author Contributions

AO-M and GL contributed to the conception and design of the study. FH, SL, and AO-M recruited the participants. FH, SL, VT, MM, M-PH, FD, and AO-M collected the data. FH, SL, VT, FD, MM, M-PH, and L-DB organized the data base. FH, SL, VT, M-PH, L-DB, and GL performed the statistical analysis and interpreted the results. FH and VT made the first draft of the paper. All authors contributed to manuscript revision, read and approved the submitted version.

## Conflict of Interest Statement

The authors declare that the research was conducted in the absence of any commercial or financial relationships that could be construed as a potential conflict of interest.

## Abbreviations

BMI: body mass index
CNS: central nervous system
cSP: contralateral silent period
CVs: coefficients of variation
EMG: electromyography
FDI: first dorsal interosseous
ICC: intraclass correlation coefficient
MEPs: motor evoked potentials
MVC: maximum voluntary contraction
M1: primary motor cortex
RC: recruitment curve
rMT: resting motor threshold
MSO: stimulator’s maximum output
SEM_eas_: standard error of the measurement
SDC_indv_: smallest detectable change for an individual
TMS: transcranial magnetic stimulation
